# Characterization of HIV Transmission in South-East Austria

**DOI:** 10.1371/journal.pone.0151478

**Published:** 2016-03-11

**Authors:** Martin Hoenigl, Antoine Chaillon, Harald H. Kessler, Bernhard Haas, Evelyn Stelzl, Karin Weninger, Susan J. Little, Sanjay R. Mehta

**Affiliations:** 1 Division of Infectious Diseases, University of California San Diego, San Diego, California, United States of America; 2 Section of Infectious Diseases and Tropical Medicine, Department of Internal Medicine, Medical University of Graz, Graz, Austria; 3 Division of Pulmonology, Department of Internal Medicine, Medical University of Graz, Graz, Austria; 4 Institute of Hygiene, Microbiology and Environmental Medicine, Medical University of Graz, Graz, Austria; 5 Department of Infectious Diseases, Landeskrankenhaus Graz West, Graz, Austria; 6 Veterans Affairs Healthcare System, San Diego, California, United States of America; Centro Nacional de Microbiología - Instituto de Salud Carlos III, SPAIN

## Abstract

To gain deeper insight into the epidemiology of HIV-1 transmission in South-East Austria we performed a retrospective analysis of 259 HIV-1 partial *pol* sequences obtained from unique individuals newly diagnosed with HIV infection in South-East Austria from 2008 through 2014. After quality filtering, putative transmission linkages were inferred when two sequences were ≤1.5% genetically different. Multiple linkages were resolved into putative transmission clusters. Further phylogenetic analyses were performed using BEAST v1.8.1. Finally, we investigated putative links between the 259 sequences from South-East Austria and all publicly available HIV polymerase sequences in the Los Alamos National Laboratory HIV sequence database. We found that 45.6% (118/259) of the sampled sequences were genetically linked with at least one other sequence from South-East Austria forming putative transmission clusters. Clustering individuals were more likely to be men who have sex with men (MSM; p<0.001), infected with subtype B (p<0.001) or subtype F (p = 0.02). Among clustered males who reported only heterosexual (HSX) sex as an HIV risk, 47% clustered closely with MSM (either as pairs or within larger MSM clusters). One hundred and seven of the 259 sequences (41.3%) from South-East Austria had at least one putative inferred linkage with sequences from a total of 69 other countries. In conclusion, analysis of HIV-1 sequences from newly diagnosed individuals residing in South-East Austria revealed a high degree of national and international clustering mainly within MSM. Interestingly, we found that a high number of heterosexual males clustered within MSM networks, suggesting either linkage between risk groups or misrepresentation of sexual risk behaviors by subjects.

## Introduction

The poor fidelity of the HIV-1 reverse transcriptase leads to significant diversity of the viral population within infected individuals and across populations over time [1]. Viral genetic sequence information can therefore be used to reconstruct transmission networks as well as the evolutionary history of extant viruses [2,3]. Phylogeography (i.e. reconstruction of the molecular evolutionary history and the spatial diffusion of a virus) can provide clues to the dynamics of viral outbreaks [4]. Integration of molecular, clinical and demographic data offers a unique opportunity to better understand the dynamics of local transmission networks [4–6] and may help to direct intervention and prevention strategies to interrupt ongoing outbreaks, such as the one in Austria.

An estimated 9,000 HIV infected individuals (approximately two-thirds men and one-third women) are currently living in Austria, of which 7–10% live in South-East Austria [7,8]. Although the majority of HIV infected individuals (over 90%) are receiving antiretroviral therapy (ART), the annual number of new HIV diagnoses in Austria has remained stable at about 400–500 new HIV diagnoses per year during the last decade [7]. A major reason for the steady rate of incident diagnoses, despite high ART-coverage, is that despite high numbers of per-capita testing (compared to other European countries), only 20% of individuals are diagnosed with acute or recent HIV infection (defined as seroconversion, with the last negative HIV test not more than 3 years before the first positive test) [7,8]. Among the most important factors associated with late HIV diagnosis (defined as CD4 cell count below 350 at time of HIV diagnosis and/or AIDS within 3 months of HIV diagnosis) are reported heterosexual (HSX) sex as the mode of HIV acquisition and residing in rural areas (i.e. population size of residence below 100,000) [8,9].

To gain deeper insight into HIV transmission, this study aimed to reconstruct the local HIV-1 transmission network in South-East Austria, representing an area of more than 1 million inhabitants, and to investigate putative links with publicly available HIV *pol* sequences from around the world.

## Materials and Methods

### Study Population

The study population included 259 residents of South-East Austria with newly diagnosed HIV-1 infection between 2008 and 2014, who had initial nucleic acid amplification testing (NAT) and/or resistance testing performed at the Institute of Hygiene, Microbiology and Environmental Medicine of the Medical University in Graz. This institute represents the only laboratory performing HIV NAT in this region of more than 1 million inhabitants.

Demographic information and clinical data were retrospectively collected at the Department of Infectious Diseases, Landeskrankenhaus Graz South-West, Austria, where the vast majority of HIV patients were linked to care, and the Department of Medicine, Medical University of Graz, Austria. Data collected included age, sex, HIV risk factor(s), and area of residence for all subjects. All demographic data, except for location of residence, was collected in a de-identified manner and then linked to the unique HIV sequence.

To analyze the geographic network characteristics of viral transmission in the area, the region of South-East Austria under investigation was first divided into five areas (each including at least 15 HIV study participants, in order to reduce the risk of re-identification of participants). These areas included: Graz (i.e. the only urban area within South-East Austria, with about 300,000 inhabitants), the suburban district Graz County (later referred to as “suburban”), and the rural districts of northern Styria, western/southern Styria, and eastern Styria (which included the southern districts of the neighboring state of Burgenland).

### Sequence Analysis and HIV Network Inference

Sequences were generated using the FDA-approved and IVD/CE-labeled TRUGENE HIV-1 Genotyping Kit (Siemens Healthcare Diagnostics, Tarrytown, NY). Analysis was performed using partial HIV-1 *pol* sequences including the regions encoding reverse transcriptase and protease. All sequences were subtyped using the Subtype Classification using Evolutionary Algorithms (SCUEAL) program [10].

All sequences were made publicly available. The GenBank accession numbers for the *pol* sequences included in this analysis are KU574281—KU574539. The study was conducted according to the principles expressed in the Declaration of Helsinki and was approved by the local ethics committee, Medical University of Graz. All data presented have been de-identified and are therefore not attributable to individual patients. At the participating centers, information of all patients admitted is automatically stored in the electronic hospital database, and written informed consent of participating patients was waived by the local ethics committee.

#### Genetic network and clustering analysis

After removing codons associated with major drug resistance mutations [11], putative transmission clusters were inferred by establishing linkage when two sequences had a Tamura-Nei 93 [12] genetic distance (D) ≤1.5%. This threshold was selected based on previous work from our group showing that within a mono-infected person, pol sequences typically do not diverge more than 1% from baseline over a decade [13], and this threshold is standard in the field [14]. Transmission clusters included all connected nodes such that all nodes (individuals) within a cluster would have a D of <1.5% from at least one other node in the cluster, but not necessarily within 1.5% of all nodes within the cluster. Demographic information and clinical data analyzed in conjunction with the inferred networks included age, sex, HIV risk factor(s) and area of diagnosis for all subjects.

Transmission clusters inferred by network analysis were confirmed using phylogenetic methods. Subtype specific maximum likelihood trees were inferred using RAxML [15] and included reference sequences from the main subtypes and CRF forms as well as a set of reference sequences from the same subtype. Clusters were confirmed if bootstrap support exceeded 80%.

#### Bayesian phylogenetic and phylogeographic analyses

Phylogenetic analyses were performed by first inferring a time-scaled maximum clade credibility tree using all 259 of the South-East Austrian HIV sequences, performed using a Bayesian Markov Chain Monte Carlo (MCMC) framework as implemented in BEAST v1.8.1 [16] with BEAGLE [17]. We used a discretized gamma distribution (GTR + 4Γ) to account for among-site rate variation. Time scales of the trees were calibrated with the sampling dates available. We specified a uncorrelated lognormal (UCLN) molecular clock with a gamma distribution prior model that allows rates to vary among the branches of the inferred phylogenies to infer the timescale of HIV evolution for each subject, with a gamma distribution prior on the mean clock rate (shape = 0.001, scale = 1000). A skygrid tree prior was used as a coalescent demographic model with time-aware smoothing [18,19]. Monte Carlo simulations were run for 100 million steps, sub-sampling parameters every 50,000 steps. Convergence of the chains was inspected using Tracer v1.6. Maximum clade credibility trees were obtained with TreeAnnotator v1.8.1 and visualized by using FigTree 1.4 [20].

In order to infer the spatiotemporal dynamics of the sampled Austrian HIV epidemic (n = 259 individuals), we performed a phylogeographic analysis in which we categorized individuals into two groups residing in either: ‘urban areas’ (defined as Graz City and suburban Graz County, n = 126) or ‘rural areas’ (including all the other surrounding rural areas, n = 133). After applying these discrete binary geographic traits (‘urban’ or ‘rural’) to each taxon, we then evaluated viral migration patterns between these regions using discrete non-reversible diffusion models and a Bayesian stochastic search variable selection (BSSVS) approach [21], as previously described [21].

#### Links between the South-East Austrian Epidemic and the Global HIV Epidemic

Finally, to better understand the intersection between the South-Eastern Austrian HIV Epidemic and the global HIV epidemic, we applied the network-based approach described above and used the HIV Trace program developed by our colleagues at UCSD to recover all putative links between the 259 sequences from Austria and all publicly available HIV polymerase sequences in the Los Alamos National Laboratory HIV sequence database (n = 148,598 sequences) [22].

### Statistical analysis

Statistical analysis was performed using SPSS, version 22 (SPSS, Inc., Chicago, IL, USA). Demographic data are displayed as absolute numbers plus percentages, medians plus interquartile ranges (IQR) or means plus 95% confidence intervals (95%CI), as appropriate. We compared demographic data, HIV risk factors, and HIV subtypes between individuals that clustered within the transmission network to those that did not cluster, by using two tailed Chi-squared or Fisher’s exact test for categorical variables, and Mann-Whitney-U test for continuous variables, as appropriate.

## Results

### Study Population

The mean age of the study population at the time of HIV diagnosis was 34 years (range 4–73 years). Eleven individuals (3.8%) were sampled during the acute stage (HIV NAT+/Ab-) of HIV infection. All individuals were ART-naïve at the time the sequences were obtained. Demographic characteristics, risk factors, and HIV subtypes are displayed in Table 1.

**Table 1:** Baseline Demographic and Viral Characteristics.

	South-East Austria 2008–2014	Individuals clustering within the network	Non-clustering individuals	*P value*
N (%)	259 (100%)	118 (46%)	141 (54%)	-
***Age (years)***				
Mean (range)	34 (4–73)	33 (17–65)	34 (4–73)	*n*.*s*.
Young (<24)	20.5% (n = 53)	20.3% (n = 24)	20.6% (n = 29)	*n*.*s*.
Older (>50)	10.4% (n = 27)	8.5% (n = 10)	12.1% (n = 17)	*n*.*s*.
***Sex***				
Male	74.5% (n = 193)	83.9% (n = 99)	66.7% (n = 94)	***0*.*002***
Female	25.5% (n = 66)	16.1% (n = 19)	33.3% (n = 47)	***0*.*002***
**Race**				
Caucasian	85% (n = 221)	93% (n = 110)	79% (n = 111)	***0*.*001***
Black	13% (n = 34)	7% (n = 8)	18% (n = 26)	***0*.*006***
Asian	2% (n = 4)	0	3% (n = 4)	***n*.*s***.
***Risk Factor***				
MSM	47.1% (n = 122)	59.3% (n = 70)	36.9% (n = 52)	***p < 0*.*001***
HSX	44.4% (n = 115)	31.4% (n = 37)	55.3% (n = 78)	***p < 0*.*001***
IDU†	6.6% (n = 17)	9.3% (n = 11)	4.3% (n = 6)	*n*.*s*.
Others/unknown	1.9% (n = 5)	0% (n = 0)	3.5% (n = 5)	*n*.*s*.
***HIV Subtype***				
B	66% (n = 171)	76.3% (n = 90)	57.4% (n = 81)	***p = 0*.*003***
CRF01_AE	12.4% (n = 32)	9.3% (n = 11)	14.9% (n = 21)	*n*.*s*.
CRF02_AG	6.9% (n = 18)	3.4% (n = 4)	9.9% (n = 14)	***p = 0*.*049***
F	5.8% (n = 15)	9.3% (n = 11)	2.8% (n = 4)	***p = 0*.*033***
C	5.0% (n = 13)	0% (n = 0)	9.2% (n = 13)	***p<0*.*001***
A	1.5% (n = 4)	0% (n = 0)	2.8% (n = 4)	*n*.*s*.
Others ^$^	2.3% (n = 6)	1.7% (n = 2)	2.8% (n = 4)	*n*.*s*.
**Residency**				
Urban	48.6% (n = 126)	49.2% (n = 58)	48.2% (n = 68)	n.s.

^†^: Category ‘IDU’ also includes men who are both MSM and IDU.

^$^: Includes G, H, J and K subtypes.

Abbreviations: CRF, circulating recombinant forms; HSX, heterosexual orientation; IDU, injection drug use; MSM, men who have sex with men; n.a., data not available; n.s., not significant.

The majority of subjects (193/259 [75%]) were male, and 122/193 (63%) of these self-identified as men who have sex with men (MSM), while 54/193 (28%) reported only heterosexual sex as risk factor for HIV acquisition. Injection drug use (IDU) was reported by 17 (9%) males (in 15 IDU was the only risk factor, 2 had also MSM risk). In one individual each, mother to child transmission and hemophilia were reported as risk factors. Of the 54 males reporting heterosexual contact as their only HIV risk, 13 (24%) reported sex with a female from a country with >1% HIV prevalence, 11 (20%) reported origin from a country with >1% HIV prevalence, and 2 (4%) reported sex with a female known to be HIV positive, while 28 (52%) could not specify their risk, other than having heterosexual contacts.

Sixty-six of 259 (25%) subjects diagnosed with HIV were female. The vast majority (61/66) reported heterosexual contact as their only risk factor for HIV acquisition, while two females reported IDU as risk factor and three reported nosocomial transmission. Of the 61 reporting heterosexual contact as risk factor, 24 (39%) reported sex with a male from a country with >1% HIV prevalence, 4 (7%) sex with an IDU male, 2 (3%) sex with a MSM, and 4 (7%) sex with a male known to be HIV positive. The remaining 27 (44%) females could not specify their risk, other than having heterosexual contacts.

Evolution of the relative frequency of the reported risk exposure among HIV individuals from the South-East Austria Cohort between 2008 and 2014 revealed that MSM and heterosexual contact were the two major risk exposures each accounting for 40%-60% of cases each year of the study period.

### Cluster Analysis

**Fig 1:**
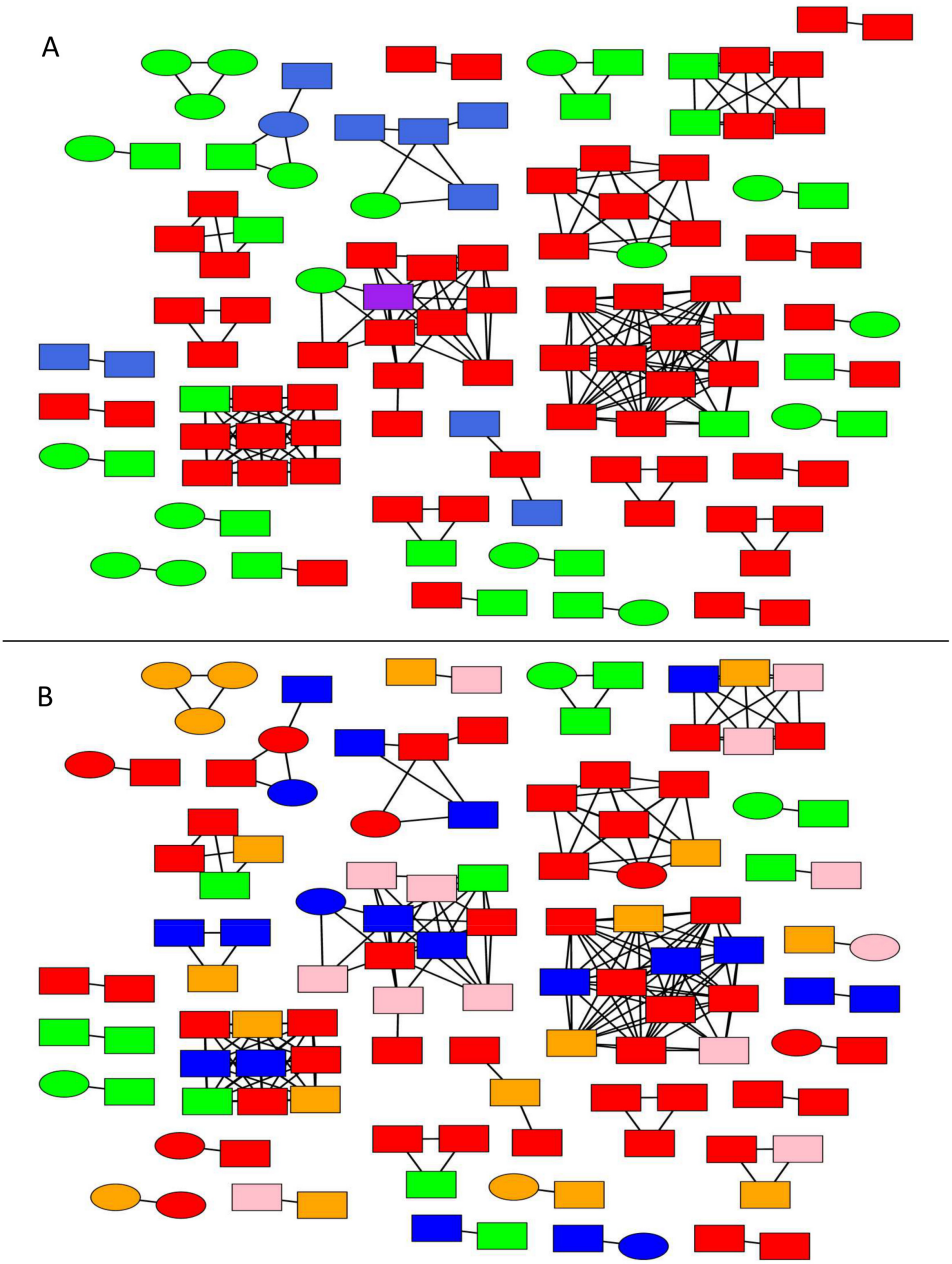
Inferred HIV Transmission Clusters. HIV-1 transmission clusters (networks) identified in the region of South-East Austria. Sex of the individuals (nodes) is indicated by shape: square (men) and circle (women) **1A**. Nodes are colored by their reported risk factor: red (men who have sex with men [MSM]), green (heterosexual), blue (injection drug use [IDU]) and purple (MSM and IDU), respectively. All edges represent a genetic distance ≤1.5% separating nodes. **1B**. Here, nodes are colored according to the region of residence in red (Graz City), pink (Suburban Graz), green (Eastern Styria), blue (Northern Styria) and orange (Southwestern Styria).

**Fig 2:**
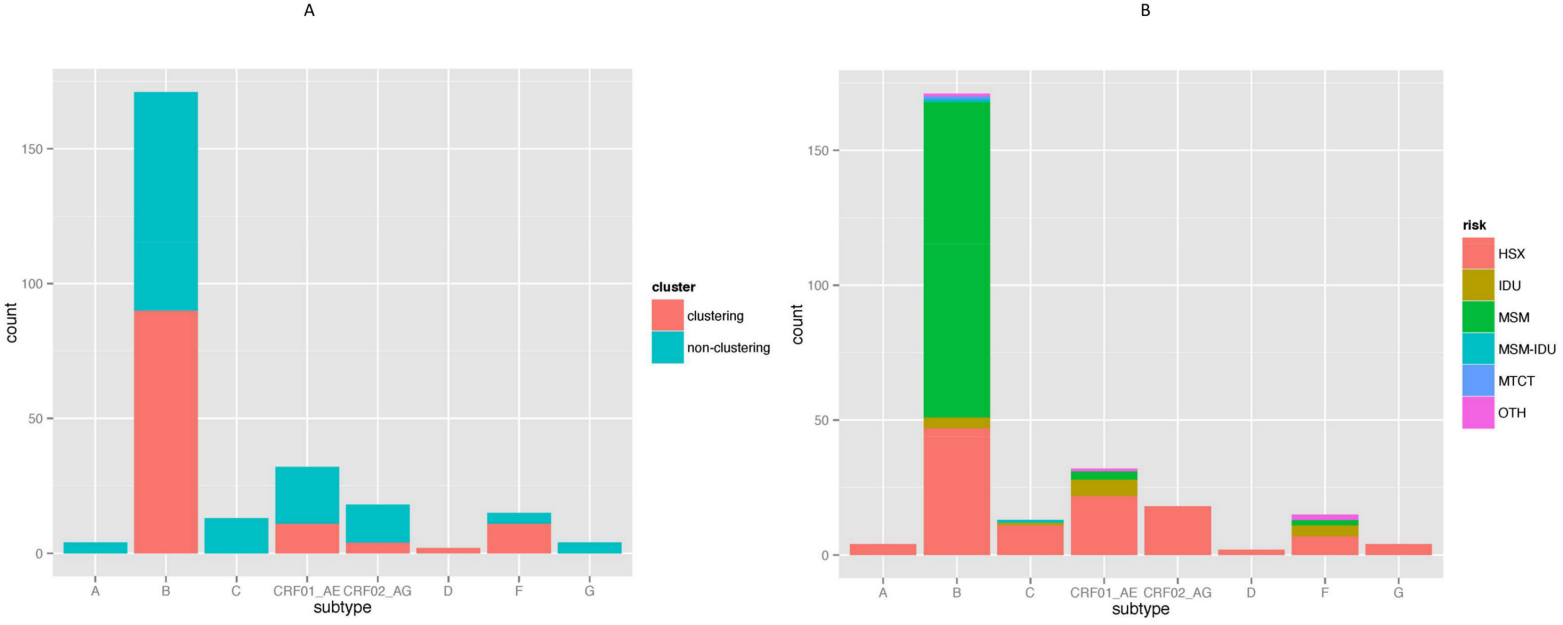
HIV Subtype in the South-East Austrian Cohort. **2A**. Comparison between clustering and non-clustering populations by HIV subtype. **2B**. HIV Subtype by Risk factors in the South-East Austrian Cohort. Legend: HSX, heterosexual contact; IDU, injection drug use; MSM, men who have sex with men; MTCT, mother to child transmission; OTH, other.

Putative transmission linkages were inferred for 45.6% (118/259) of the sequences from South-East Austria (Fig 1). The likelihood of clustering for an individual did differ by sex (p = 0.002, males more likely to cluster), race (Caucasians more likely to cluster, p = 0.001; Blacks less likely to cluster, p = 0.006) but not by area of residence type (urban versus rural; Table 1). Clustering individuals were more likely to report MSM as their major risk factor (p<0.001) than the individuals who did not belong to any detected cluster in this dataset. A higher prevalence of subtype B (p<0.001) and F (p = 0.033) was found in clustering individuals (Table 1, Fig 2a). HIV subtypes by risk behavior are displayed in Fig 2b. We found a significantly higher proportion of MSM among individuals infected by subtype B (p<0.001) and a higher proportion of heterosexuals (p<0.001) and IDU (p = 0.002) among non-B individuals.

**Fig 3:**
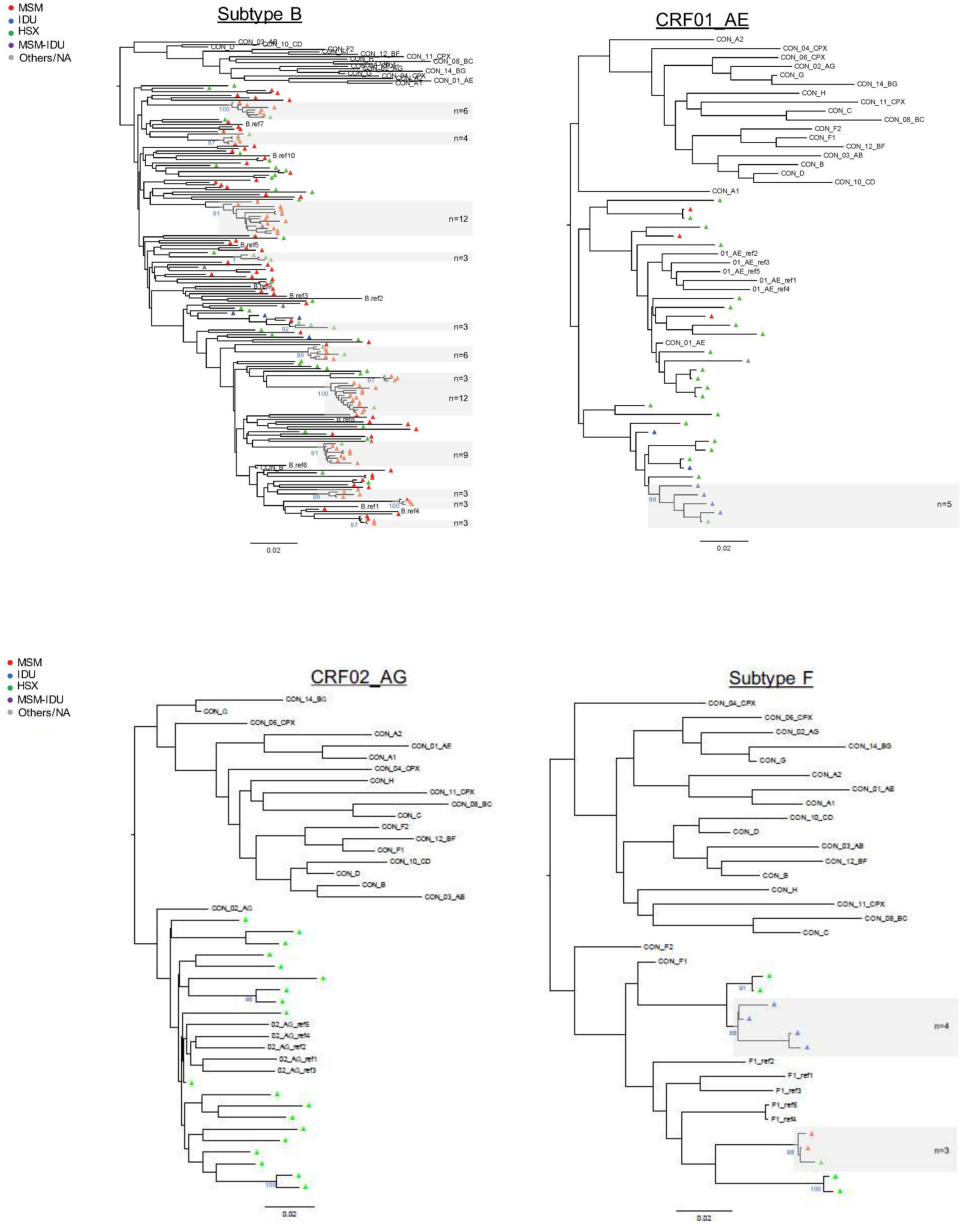
Maximum likelihood phylogenetic trees (by subtype). All clusters identified by network analysis were supported by a bootstrap value above 80%. The bootstrap value of the larger clusters is indicated in blue at the root of each corresponding clusters and non-dyad clusters are highlighted in grey with the cluster size indicated for each cluster. Tips are colored by their reported risk factor in red (men who have sex with men, MSM), green (heterosexual, HSX), blue (injection drug use, IDU), purple (MSM plus IDU) and grey (unknown/others), respectively. All trees (Subtype B, CRF_01AE, CRF_02AG and subtype F) include a set of reference consensus sequences from the main subtypes and CRF forms, and a set of reference sequences from the same subtype.

A total of 34 clusters were identified (Fig 1, Table 2). 19 of these clusters (55.9%) were linked pairs, while the remaining 15 clusters (44.1%) had three or more individuals, including two clusters each with 12 individuals. MSM were more likely to be members of larger clusters (>2 individuals) than smaller clusters (p = 0.028) (Table 2, Fig 1a). Three of the four transmission clusters that included IDU were non-B clusters, including two subtype F and one CRF01_AE cluster (Fig 1). Maximum likelihood trees for each subtype were generated to validate the clusters identified by the network inference method above (Fig 3).

**Table 2:** Characteristics of clusters according to cluster size.

Cluster Size	Pairs (n = 19)	Triplets (n = 7)	≥4 individuals (i.e. large clusters) (n = 8)
***Age*** *(median*, *range)*	36 (22–64)	31 (22–40)	33 (25–53)
***Risk Factor***			
HSX exclusive	42.1% (n = 8)	28.6% (n = 2)	0
MSM exclusive	31.6% (n = 6)	42.9% (n = 3)	0
Mixed MSM/HSX	21.1% (n = 4)	14.3% (n = 1)	75% (n = 6)
IDU†	5.3% (n = 1)	14.3% (n = 1)	25% (n = 2)
***Subtype***			
B	57.9% (n = 11)	85.7% (n = 6)	75% (n = 6)
CRF01_AE	15.8% (n = 3)	0% (n = 0)	12.5% (n = 1)
CRF02_AG	10.5% (n = 2)	0% (n = 0)	0
F	10.5% (n = 2)	14.3% (n = 1)	12.5% (n = 1)
D	5.2% (n = 1)	0% (n = 0)	0

^†^ Category ‘IDU’ also includes men who are both MSM and IDU.

Abbreviations: HSX, heterosexual; IDU, injection drug use; MSM, men who have sex with men.

Of the clustering males that reported only HSX risk, 47% (9/19) clustered closely with MSM. Of these nine, seven reported that they were certain about heterosexual transmission without giving further information on risk, while two reported sex with a female from a high prevalence country. Five out of those nine males clustered within the four large MSM clusters. While one of these clusters included four MSM and two HSX males, the remaining three HSX males were the only non-MSM within their respective transmission clusters. The remaining four of nine males reporting HSX contact clustered as pairs (n = 3) or triplets (n = 1) with MSM (Fig 1a).

Using Bayesian phylogenetic methods, we then inferred the time of most recent common ancestor (tMRCA) for the three largest clusters. These three clusters, which were all subtype B and included 9, 11 and 12 individuals respectively, had tMRCAs of 2006 (95% Confidence Interval [CI] 2001–2011), 1998 (95%CI 1992–2003) and 1999 (95%CI:1993–2003) respectively.

### Migration Analysis

**Fig 4:**
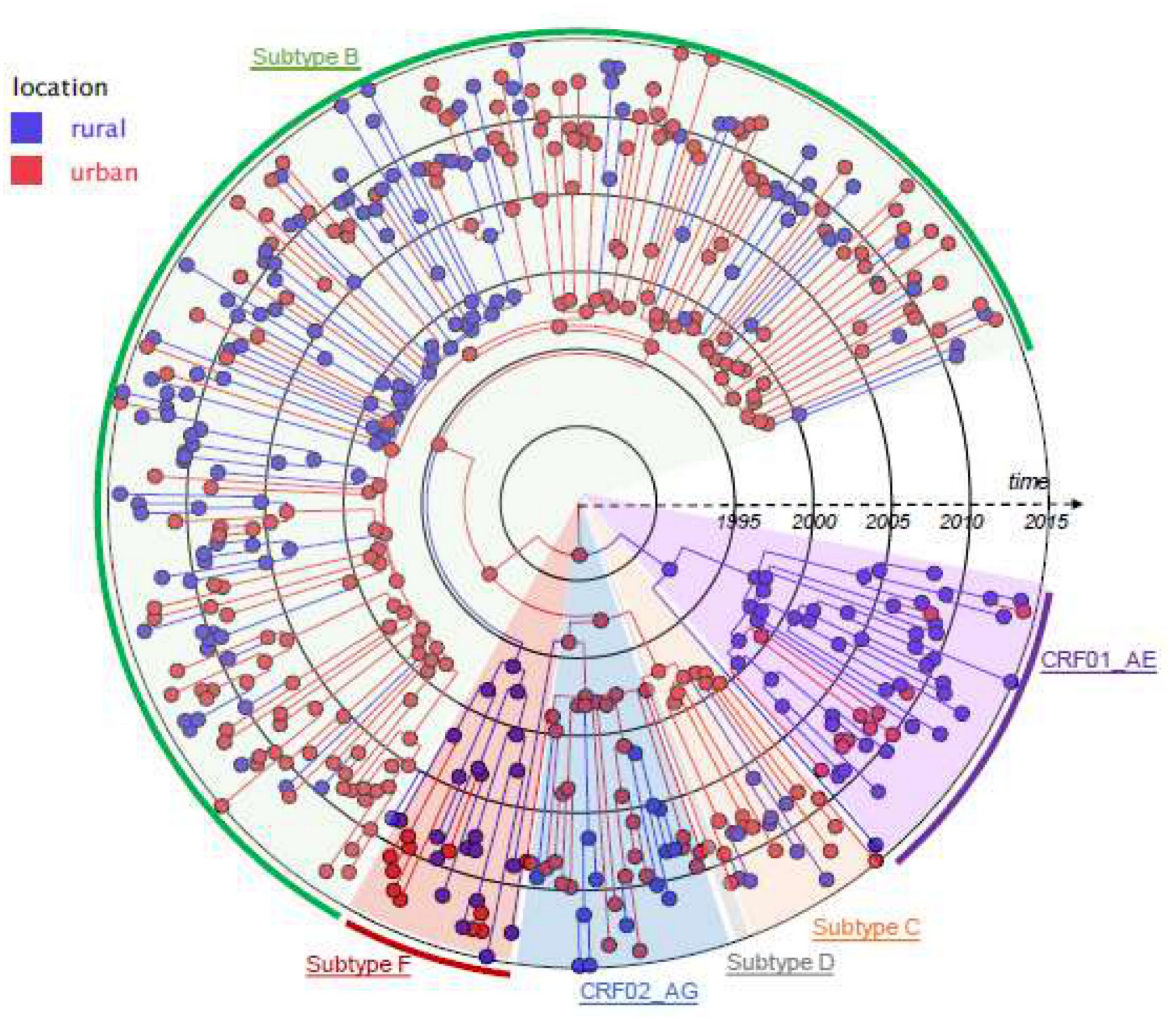
Time-scaled phylogeographic reconstruction of the 259 HIV-1 sequences from South-East Austria. Ancestral nodes are colored according to the inferred most probable location state with the color codes corresponding to the urban areas (Graz City and suburban Graz) in red and the rural areas in blue. Branch colors represent the most probable location of the parental node of each branch. Tips are colored according to the observed sampling location of each sequence. Each clade is highlighted in unique color: green (subtype B), red (subtype F), blue (CRF02_AG), gray (subtype D), orange (Subtype C) and purple (CRF01_AE).

Based on visual inspection (Fig 1b), there was no clear relationship between clustering individuals and their area of residence, suggesting a high degree of geographic mixing of the population within South-East Austria. To further examine the relationship between geography and HIV transmission, we next investigated the spatio-temporal dynamics of the observed epidemic specifically between urban and rural regions of South East Austria by applying a Bayesian phylogeographic inference using discrete non-reversible models to our geo-referenced sequences. In this phylogeographic reconstruction, each node was annotated with a geographic location (Fig 4). The resulting phylogeny demonstrates inferred ancestral nodes from either rural (in blue) or urban areas (in red). Branches linking red and blue nodes represent location state transitions (viral lineage migrations) between the rural and urban regions. We found that the sampled subtype B HIV epidemic was mainly initiated in the urban areas of Graz (in red) as illustrated by the most probable location state of the descendant nodes with 57.1% (95%CI 54%-60.1%) of all viral lineages movements originating from urban Graz (Fig 4). For the HIV-1 subtype F and CRF01_AE epidemic, all of the sampled viruses and the inferred ancestral nodes corresponded to rural areas.

#### Intersection between the Global HIV Epidemic and South East Austria

**Fig 5:**
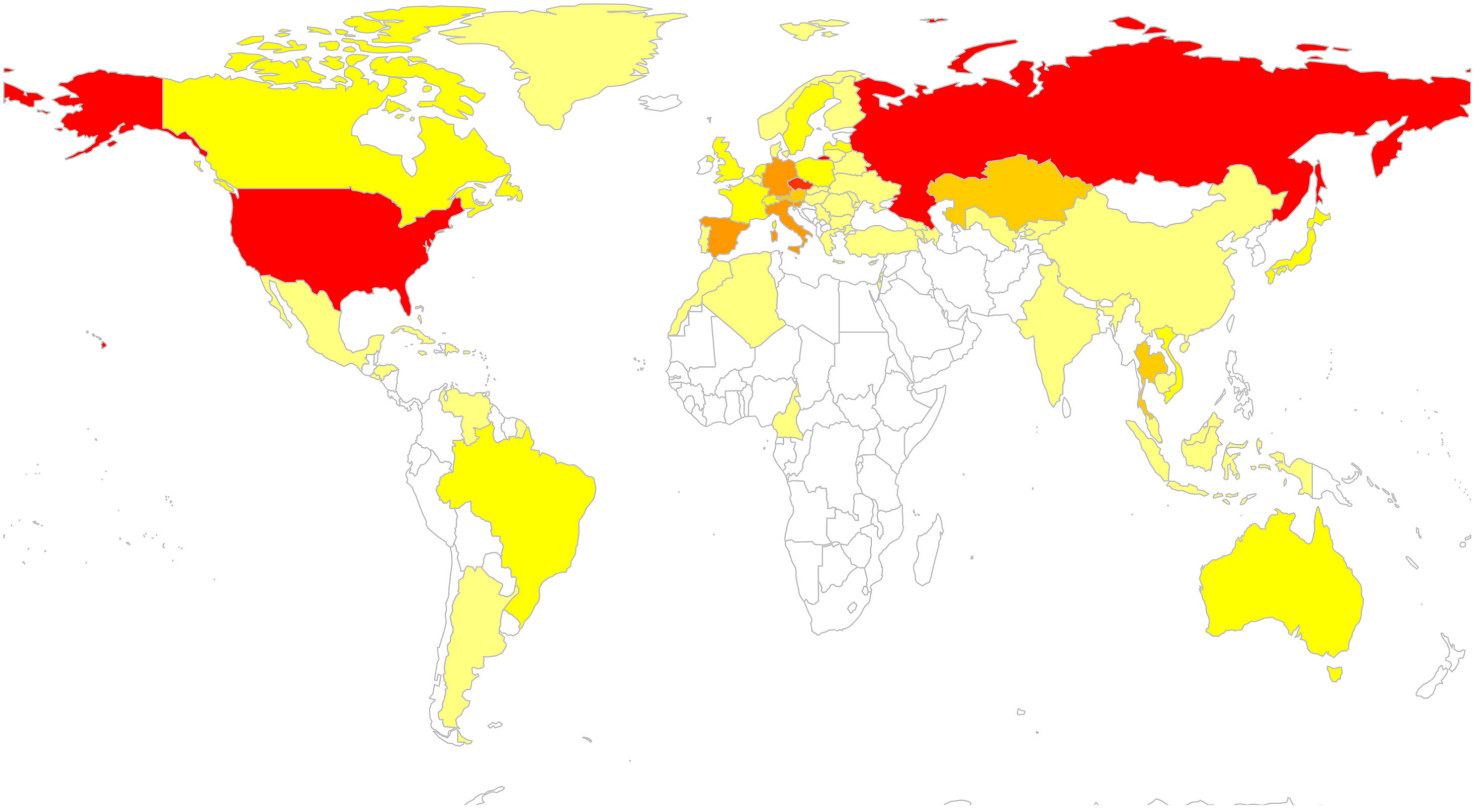
Intersection between the Global HIV Type 1 Endemic and South East Austria. A total of 107 (41.3%) of 259 sequences obtained in South-East Austria shared a putative link with sequences from foreign countries/regions. In this heat map with a gradient from 1 to 40 linkages, countries are colored by the number of putative links from yellow to red (i.e. red is 40 or more linkages). Unlinked countries are colored in white.

One hundred and seven of the 259 sequences (41.3%) from this collection had a putative inferred linkage with a sequence from outside Austria. These sequences were linked with sequences from 69 other countries. The 14 Austrian CRF01_AE sequences who had a putative inferred link to a non-Austrian sequence, were linked to 158 CRF01_AE sequences (predominantly from southeast Asia), and 1215 subtype A sequences (predominantly from large transmission clusters from Eastern Europe) (Fig 5). The majority of linked sequences were subtype B (76%), and originated from MSM (64%) (S1 Fig).

## Discussion

We reconstructed the HIV-1 transmission network in South-East Austria by analyzing 259 sequences of persons newly diagnosed with HIV-infection between 2008 and 2014. Network analysis revealed a high rate of inferred clustering within the cohort, consisting mainly of MSM and injection drug users, and a large number of international connections.

The sequences included in this analysis represent an estimated 7.4% of the new HIV diagnoses across Austria in this time period, and over 95% of the new HIV diagnoses in South-East Austria [7,8]. We were therefore able to reconstruct a relatively complete network of individuals newly diagnosed with HIV in South-East Austria between 2008 and 2014. We found a high degree of clustering, with putative transmission linkages for 45.6% (118/259) of the sequences.

Nearly a third of HIV positive males identified HSX contact as their only risk for HIV acquisition, which may be considered a high proportion when compared to other settings in Europe and the United States [23–27]. Interestingly, of the clustering males that reported only HSX risk, 47% (9/19) clustered closely with MSM (either as pairs or within larger MSM clusters). This suggests misrepresentation of risk that may be related to perceived stigma in South-East Austria that prevents males from self-identifying as MSM, or may be related to a group of “missing” individuals with two risk factors (i.e. HSX/MSM [Bisexual], or MSM/IDU) bridging these individuals to MSM HIV-1 transmission networks. Local transmission networks of HIV infected persons are never complete because i) sampling of the entire network is often impossible, such that the inferred network will not include HIV-infected persons not yet diagnosed. Further, a putatively inferred linkage does not represent a true transmission link, as additional unsampled individuals could have been members of the transmission chain linking the two observed putatively linked individuals [2]. Therefore we can’t rule out that there might be”missing” individuals bridging our sampled HIV-1 infected MSM and HSX males. Our results correlate well with characteristics of the overall Austrian HIV epidemic. Interestingly heterosexual contact was the most frequently reported mode of HIV acquisition until 2008, and only recently surpassed by men who sex with men (MSM) sexual contact [8]. In part, these findings may be explained by stigma and discrimination that may limit individuals from self-identifying as MSM. Austria, while influenced by Roman Catholicism, has slowly become more liberal with laws and social opinions concerning sexual orientation and gender identity. Lesbian, gay, bisexual, and transgender (LGBT) persons in Austria are, however, still facing some legal challenges not experienced by non-LGBT residents [28]. In a survey conducted by the European Union in 2012, collecting online-information from over 93,000 LGTB persons, 48% of respondent from Austria felt discriminated against or harassed on the grounds of sexual orientation in the last 12 months with an average number of 1,078 harassment incidents per 1000 respondents [28]. More than that, only 26% of Austrian LGBT respondents reported that they reveal their sexual orientation or gender identity to most people in their private and professional lives, while 29% reported that they never reveal their LGBT lifestyle [28].

Bayesian phylogeographic analyses also provided insights into the spatiotemporal dynamics of the South-East Austrian HIV epidemic, suggesting a key role for the densely populated region of Graz as a hub for transmission of new HIV infections throughout the region. These results are consistent with our recent evaluation of French individuals with primary HIV infection which revealed an early spread of the HIV epidemic from Paris to the rest of the Country [29]. Interestingly, these analyses also showed that the subtype F and CRF01_AE local epidemics were more likely to have started (and persisted) in rural areas (Fig 4).

Previously, Frentz and co-workers evaluated 1,330 clustering pol sequences from 26 European countries collected between 2002 and 2007 and found that transmission of HIV-1 in Europe is predominantly occurring between individuals from the same country as only 25.8% of sequences clustered with individuals from foreign countries (10.2% with individuals from neighboring countries only and 15.6% with individuals from countries without a common border) [28]. In contrast, our study found that more than 40% of sequences had putative linkages with sequences from countries outside Austria, with the majority of linkages to Eastern European countries without a common border. The main explanation for these differing findings can be found in the size of the network tested. While Frentz and colleagues evaluated linkage within a limited pool of 4,260 pol sequences only, our study evaluated linkage using nearly 150,000 sequences of the Los Alamos National Laboratory HIV sequence database. In contrast to Frentz et al., our findings may therefore indicate that infections through travelling between countries may be more frequent and a more international approach that is not restricted to national borders is needed for surveillance of HIV-1 epidemics.

In conclusion, analysis of HIV1 sequences from South-East Austria revealed a highly clustered transmission network with a large number of international connections. Higher rates of clustering in MSM, and a high proportion of HSX males clustering within MSM networks may inform prevention, testing and linkage to care strategies. Future studies are needed to further expand that network to other parts of Austria and other countries across Europe.

## Supporting Information

S1 Fig Characteristics of the 107 sequences from the South-Eastern Austrian Cohort putatively linked with sequences from foreign countries/regions by A. Subtype, and B. Risk Factor. HSX: heterosexual; MSM: Men who have sex with men; IDU: Injection Drug Users.
(PDF)

## References

[1] Holmes EC. Error thresholds and the constraints to RNA virus evolution. Trends Microbiol. 2003;11: 543–546. 14659685 10.1016/j.tim.2003.10.006 PMC7172642

[2] Mehta SR, Wertheim JO, Delport W, Ene L, Tardei G, Duiculescu D, et al Using phylogeography to characterize the origins of the HIV-1 subtype F epidemic in Romania. Infect Genet Evol. 2011;11: 975–979. 10.1016/j.meegid.2011.03.009 21439403 PMC3104099

[3] Little SJ, Kosakovsky Pond SL, Anderson CM, Young JA, Wertheim JO, Mehta SR, et al Using HIV Networks to Inform Real Time Prevention Interventions. PLoS One. 2014;9: e98443 10.1371/journal.pone.0098443 24901437 PMC4047027

[4] Lemey P, Rambaut A, Drummond AJ, Suchard MA. Bayesian phylogeography finds its roots. PLoS Comput Biol. 2009;5: e1000520 10.1371/journal.pcbi.1000520 19779555 PMC2740835

[5] Dennis AM, Hue S, Hurt CB, Napravnik S, Sebastian J, Pillay D, et al Phylogenetic insights into regional HIV transmission. AIDS. 2012;26: 1813–1822. 22739398 10.1097/QAD.0b013e3283573244 PMC3566771

[6] Bello G, Guimaraes ML, Morgado MG. Evolutionary history of HIV-1 subtype B and F infections in Brazil. AIDS. 2006;20: 763–768. 16514307 10.1097/01.aids.0000216377.84313.52

[7] UNAIDS. Austria: Country Progress Report 2012. 2012.

[8] Gisinger M, Kitchen M, Leierer G, Rappold M, Sarcletti M, Strickner S, et al. 26th Report of the Austrian HIV Cohort Study. 2014;26.

[9] Salzer HJ, Hoenigl M, Kessler HH, Stigler FL, Raggam RB, Rippel KE, et al Lack of risk-awareness and reporting behavior towards HIV infection through needlestick injury among European medical students. Int J Hyg Environ Health. 2011;214: 407–410. 10.1016/j.ijheh.2011.05.002 21665538

[10] Kosakovsky Pond SL, Posada D, Stawiski E, Chappey C, Poon AF, Hughes G, et al An evolutionary model-based algorithm for accurate phylogenetic breakpoint mapping and subtype prediction in HIV-1. PLoS Comput Biol. 2009;5: e1000581 10.1371/journal.pcbi.1000581 19956739 PMC2776870

[11] Lewis F, Hughes GJ, Rambaut A, Pozniak A, Leigh Brown AJ. Episodic sexual transmission of HIV revealed by molecular phylodynamics. PLoS Med. 2008;5: e50 10.1371/journal.pmed.0050050 18351795 PMC2267814

[12] Tamura K, Nei M. Estimation of the number of nucleotide substitutions in the control region of mitochondrial DNA in humans and chimpanzees. Mol Biol Evol. 1993;10: 512–526. 8336541 10.1093/oxfordjournals.molbev.a040023

[13] Hightower GK, May SJ, Perez-Santiago J, Pacold ME, Wagner GA, Little SJ, et al HIV-1 clade B pol evolution following primary infection. PLoS One. 2013;8: e68188 10.1371/journal.pone.0068188 23840830 PMC3695957

[14] Aldous JL, Pond SK, Poon A, Jain S, Qin H, Kahn JS, et al Characterizing HIV transmission networks across the United States. Clin Infect Dis. 2012;55: 1135–1143. 22784872 10.1093/cid/cis612 PMC3529609

[15] Stamatakis A. Using RAxML to Infer Phylogenies. Curr Protoc Bioinformatics. 2015;51: 6.14.1–6.14.14. 10.1002/0471250953.bi0614s51 26334924

[16] Drummond AJ, Rambaut A. BEAST: Bayesian evolutionary analysis by sampling trees. BMC Evol Biol. 2007;7: 214 17996036 10.1186/1471-2148-7-214 PMC2247476

[17] Ayres DL, Darling A, Zwickl DJ, Beerli P, Holder MT, Lewis PO, et al BEAGLE: an application programming interface and high-performance computing library for statistical phylogenetics. Syst Biol. 2012;61: 170–173. 10.1093/sysbio/syr100 21963610 PMC3243739

[18] Drummond AJ, Nicholls GK, Rodrigo AG, Solomon W. Estimating mutation parameters, population history and genealogy simultaneously from temporally spaced sequence data. Genetics. 2002;161: 1307–1320. 12136032 10.1093/genetics/161.3.1307 PMC1462188

[19] Minin VN, Bloomquist EW, Suchard MA. Smooth skyride through a rough skyline: Bayesian coalescent-based inference of population dynamics. Mol Biol Evol. 2008;25: 1459–1471. 10.1093/molbev/msn090 18408232 PMC3302198

[20] Drummond AJ, Suchard MA, Xie D, Rambaut A. Bayesian phylogenetics with BEAUti and the BEAST 1.7. Mol Biol Evol. 2012;29: 1969–1973. 10.1093/molbev/mss075 22367748 PMC3408070

[21] Muniraju M, Munir M, Parthiban AR, Banyard AC, Bao J, Wang Z, et al Molecular evolution of peste des petits ruminants virus. Emerg Infect Dis. 2014;20: 2023–2033. 10.3201/eid2012.140684 25418782 PMC4257836

[22] Kuiken C, Korber B, Shafer RW. HIV sequence databases. AIDS Rev. 2003;5: 52–61. 12875108 PMC2613779

[23] Hoenigl M, Weibel N, Mehta SR, Anderson CM, Jenks J, Green N, et al Development and Validation of the San Diego Early Test (SDET) Score to Predict Acute and Early HIV Infection Risk in Men who have Sex with Men. Clin Infect Dis. 2015;61: 468–475. 10.1093/cid/civ335 25904374 PMC4542926

[24] Hoenigl M, Green N, Mehta SR, Little SJ. Risk Factors for Acute and Early HIV Infection Among men who have sex with men (MSM) in San Diego, 2008–2014: a cohort study. Medicine. 2015;94:e1830. 26469928 10.1097/MD.0000000000001830 PMC4616789

[25] Hoenigl M, Anderson CM, Green N, Mehta SR, Smith DM, Little SJ. Repeat HIV-testing is associated with an increase in behavioral risk among men who have sex with men: a cohort study. BMC Med. 2015;13: 218-015-0458-5. 10.1186/s12916-015-0458-5 PMC4596465 26444673

[26] Hoenigl M, Green N, Camacho M, Gianella S, Mehta SR, Smith DM, et al Signs or Symptoms of Acute HIV Infection in a Cohort Undergoing Community-Based Screening. Emerg Infect Dis. 2016;22: 532–534. 26890854 10.3201/eid2203.151607 PMC4766914

[27] Hoenigl M, Graff-Zivin J, Little SJ. Costs per Diagnosis of Acute HIV Infection in Community-based Screening Strategies: A Comparative Analysis of Four Screening Algorithms. Clin Infect Dis. 2016;62: 501–511. 10.1093/cid/civ912 26508512 PMC4725382

[28] European Union Agency for Fundamental Rights. European Union lesbian, gay, bisexual and transgender survey. 2014. 23787083

[29] Chaillon A, Essat A, Frange P, Barin F, Ghosn J:S, D.M., Rouzioux C, et al. Spatiotemporal Dynamics of HIV-1 Transmission in France, 1999–2014. Conference on Retroviruses and Opportunistic Infections (CROI) 2016. 2016;Abstract 222.

