# Supplementary figures and images for: Characterization of HIV Transmission in South-East Austria
Source: PLoS One. 2016 Mar 11;11(3):e0151478. doi: 10.1371/journal.pone.0151478 (PMC4788428; doi:10.1371/journal.pone.0151478)

### A. Subtype

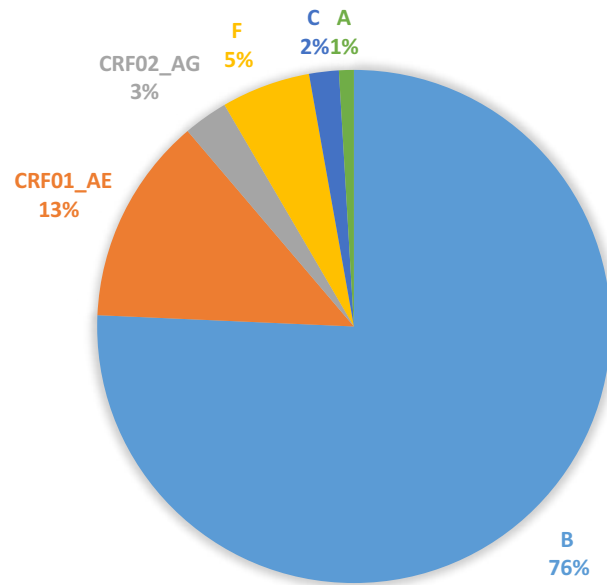

### B. Risk Behavior

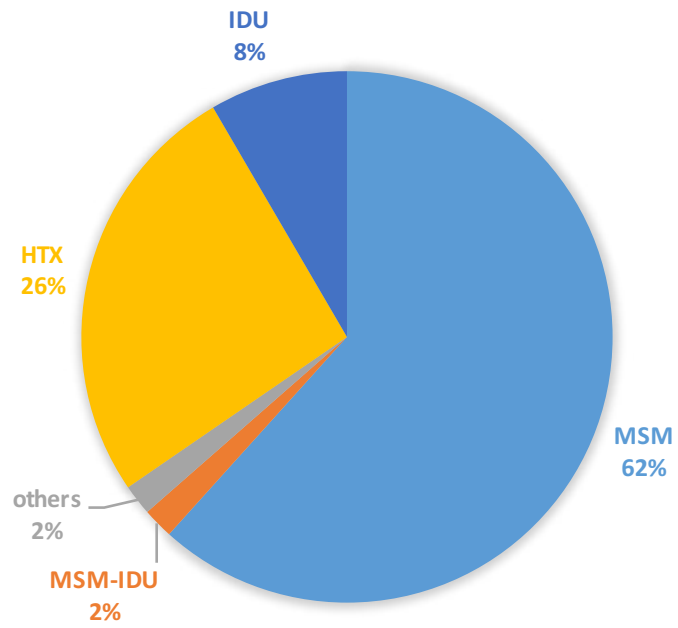

Supplement: S1 Fig — HSX: heterosexual; MSM: Men who have sex with men; IDU: Injection Drug Users. (PDF) [file pone.0151478.s001.pdf]
